# Global assessment of early warning signs that temperature could undergo regime shifts

**DOI:** 10.1038/s41598-018-28386-x

**Published:** 2018-07-03

**Authors:** Mathieu Chevalier, Gaël Grenouillet

**Affiliations:** 1 CNRS, Université Toulouse III Paul Sabatier, ENFA; UMR5174 EDB (Laboratoire Évolution & Diversité Biologique), 118 route de Narbonne, F-31062 Toulouse, France; 2 Department of Ecology, Swedish University of Agricultural Sciences, Box 7044, 750 07, Uppsala, Sweden; 3 Institut Universitaire de France, Paris, France

## Abstract

Climate change metrics have been used to quantify the exposure of geographic areas to different facets of change and relate these facets to different threats and opportunities for biodiversity at a global scale. In parallel, a suite of indicators have been developed to detect approaching transitions between alternative stable states in ecological systems at a local scale. Here, we explore whether particular geographic areas over the world display evidence for upcoming critical transitions in the temperature regime using five Early Warning Indicators (EWIs) commonly used in the literature. Although all EWIs revealed strong spatial variations regarding the likelihood of approaching transitions we found differences regarding the strength and the distribution of trends across the world, suggesting either that different mechanisms might be at play or that EWIs differ in their ability to detect approaching transitions. Nonetheless, a composite EWI, constructed from individual EWIs, showed congruent trends in several areas and highlighted variations across latitudes, between marine and terrestrial systems and among ecoregions within systems. Although the underlying mechanisms are unclear, our results suggest that some areas over the world might change toward an alternative temperature regime in the future with potential implications for the organisms inhabiting these areas.

## Introduction

Across the past decade, several studies have used metrics of climate change to quantify the exposure of geographic areas to different facets of change and relate these facets to different threats and opportunities for biodiversity^1–3^. The originality of this approach lies in the fact that it relies on climatic data with high spatio-temporal resolution (e.g. HadISST dataset) to generate “risk evaluation maps” for various biodiversity components^4^. For instance, climate velocities have been used as a surrogate to describe the migration speed required for species to keep pace with climate change and avoid extinction^3^. Those maps are useful because they make it possible to point to areas where threats and extinction risks are important and to draw inferences for unknown or poorly described species which represent most of Earth’s biodiversity^4^. However, a general assumption behind this approach is that species (or any other biodiversity component) respond in a smooth and gradual way to environmental changes, whereas there is increasing evidence that ecological systems can display non-linear and abrupt responses to such changes^5^.

Although human activities are indeed exposing biodiversity and ecosystems to gradual changes such as climate change, nutrient loading, habitat fragmentation or biotic exploitation^5,6^, recent empirical^7,8^ and theoretical^9,10^ studies have revealed that these gradual changes may induce non-linear transitions where a system shifts from one stable state to another. Such critical transitions, or regime shifts, have been highlighted not only in systems such as lakes^11^, coral reefs^12^ or forests^13^, but also in animal populations^14^ as well as in a wide spectrum of complex systems including physiological systems, financial markets and human societies^5^. These critical transitions have important social and economic implications because ecosystem state shifts can cause large changes of ecological and economic resources, and restoring a desired state may require drastic and expensive interventions^15^. Furthermore, once a tipping (or bifurcation) point (i.e. the point where the system shifts from one state to another) is crossed, a system may display hysteresis, implying that returning back to the previous state requires restoring conditions to a state well before the point where the transition occurred^5^. Detecting critical transitions before they occur is therefore paramount as it may avoid spending human and economic resources into costly restoration plans.

In general, as complex systems such as the climate or ecosystems (e.g. a lake) approach a tipping point, their dynamics tend to become dominated by a phenomenon known as critical slowing down (CSD) where the system becomes increasingly slow in recovering from perturbations^6,16^. As a result, systems approaching critical transitions tend to display common features at the vicinity of tipping point that are statistically tractable. For instance, the temporal autocorrelation^17^ and the variability of the system have been shown to increase before critical transitions^17,18^. Although CSD has been demonstrated in a variety of systems, another phenomenon, called flickering, has been proposed to characterize approaching transitions in highly stochastic environments where systems can shift to alternative basins of attraction far from tipping points^10,19^. Systems subject to this phenomenon have also been shown to display a rise variance and autocorrelation at the vicinity of tipping points in ways that resemble the effects of CSD^20^. Furthermore, because in the case of flickering external perturbations can push the system toward values that are close to the boundary between two alternative states, the asymmetry of fluctuations can change before critical transitions^21^, leading to a change in the skewness and the kurtosis of the system^10^. Overall, the return rate, the temporal autocorrelation, the variability, the skewness and the kurtosis are all expected to change as a system is approaching a critical transition and have collectively been referred to as generic early warning signals or indicators (EWIs) of regime shifts^6^.

The use of EWIs as tools to detect tipping points has recently been challenged by studies showing either that catastrophic transitions can occur without EWI (i.e. false negatives)^22^ or that EWI can occur in systems not experiencing catastrophic transitions (i.e. false positives)^23^. False positives can arise if there are changes in the stochastic regime of perturbations (e.g. increase in variability)^24^ or if the system has experienced a smooth transition toward a new and more dynamic state (e.g. a non-point attractor)^25^. Alternatively, false negatives can emerge because of data limitations (e.g. sparse or short time series), process or observation errors, non-local bifurcations or multiple noise effects^19^. Considering several EWIs has been proposed as a solution to mitigate the problem of false negatives^16^. For instance, in 14 of the 16 time series analyzed by ref.^26^, known tipping points were detected by at least one of the four EWIs considered. However, the problem with this approach it that the rate of false positives is likely to increase because considering several EWIs increases the probability to detect significant effects (increase of type I error rate). An alternative and potentially more powerful approach may be the combination of several EWIs into a single composite metric, which could allow approaching critical transitions to be more reliably inferred^27^. For instance, by combining trends in different EWIs into a single composite EWI, ref.^8^ found better estimates of an approaching transition in populations exposed to deteriorating environmental conditions relative to estimates using individual trends. Furthermore, because the signal in the composite EWI is weighted by the magnitude and the sign of individual trends, it can either reinforce or weaken the signal toward an approaching transition, with the consequence that the rate of both false positives and false negatives can be reduced. For instance, in systems where several EWIs are expected to increase e.g.^28,29^, the use of a composite EWI is particularly useful because if all individual trends are positives, then the trend in the composite EWI would also be positive and would provide further support for upcoming critical transitions. In contrast, if individual trends have different signs or magnitudes, the signal in the composite EWI would be weakened and provide lower support for approaching transitions.

One condition for critical transitions to be detected is that an underlying variable gradually pushes the system toward a tipping point^5,16^. This variable can have several origins (e.g. climate, inputs of nutrient, habitat fragmentation, species loss) and the likelihood that it triggers a catastrophic shift likely depends on the spatial and temporal scale of the study. Indeed, depending on the scale considered, a variable can either be considered as the one triggering the shift or as the one undergoing the shift. For instance, some studies e.g. ref.^30^ have considered climatic variables such as temperature as the underlying variable triggering a shift in some ecosystem states. However, temperature is itself dependent on other variables such as greenhouse gases^31^, the concentrations of which have gradually increased across the past decades^32,33^. Beyond greenhouse gases, many areas over the world have experienced strong and gradual changes in land-use (e.g. conversion of grasslands to croplands, deforestation) and land-cover (e.g. ice melting) and several studies have shown that such changes could have strong effects on some climatic variables^34^. This is because surface energy budgets are strongly influenced by the bio-geophysical characteristics of the land surface controlling atmospheric exchanges of moisture and energy. Through forestry and agricultural activities, humans perturb these characteristics, with impacts on the surface energy balance, and in turn, on local and regional temperatures^35^. For instance, changes in forest cover have been shown to affect local temperature by modifying the land-atmosphere fluxes of energy and water^36^. Given gradual changes in greenhouse gases concentration as well as land-use and land-cover, one may ask whether those changes can trigger sudden shifts in temperature and whether these shifts can be detected using EWIs.

Here we used monthly temperature time series from 1950 to 2014 collected over the world for terrestrial and marine systems at a 0.5° and 1° resolution, respectively, to investigate whether air and sea surface temperatures show any evidence of approaching a critical transition. More specifically, we computed temporal trends in five EWIs classically used in the literature (the first order autoregressive coefficient, the standard deviation, the coefficient of variation, the skewness, and the kurtosis; see the methods for further details) for 99944 pixels; 67325 of which are terrestrial and 32619 of which are marine. From those trends, we generated “risk evaluation maps” to highlight whether particular areas over the world display evidence for upcoming critical transitions and to determine whether the signal and the spatial patterns highlighted were congruent among EWIs. Although the five EWIs may describe the influence of two different phenomena (i.e. CSD and flickering), they have all been shown to behave similarly (i.e. to increase) at the vicinity of tipping points^20,21^. Thus, in a second step, we built a composite metric (see the methods) using the five above-mentioned EWIs to highlight areas showing a strong congruency among EWIs and therefore a strong probability of upcoming critical transitions. Using this composite metric, we further investigated whether trends varied across latitudes, between terrestrial and marine systems or among ecoregions within systems. Given large spatial variation in greenhouse gases concentration, land-use and land-cover changes over the world, we expected large spatial variation in EWIs. To the best of our knowledge, this study is the first using EWIs of regime shift in a prospective way to describe spatial patterns of upcoming critical transition in the temperature regime at the worldwide scale.

## Results

**Figure 1:**
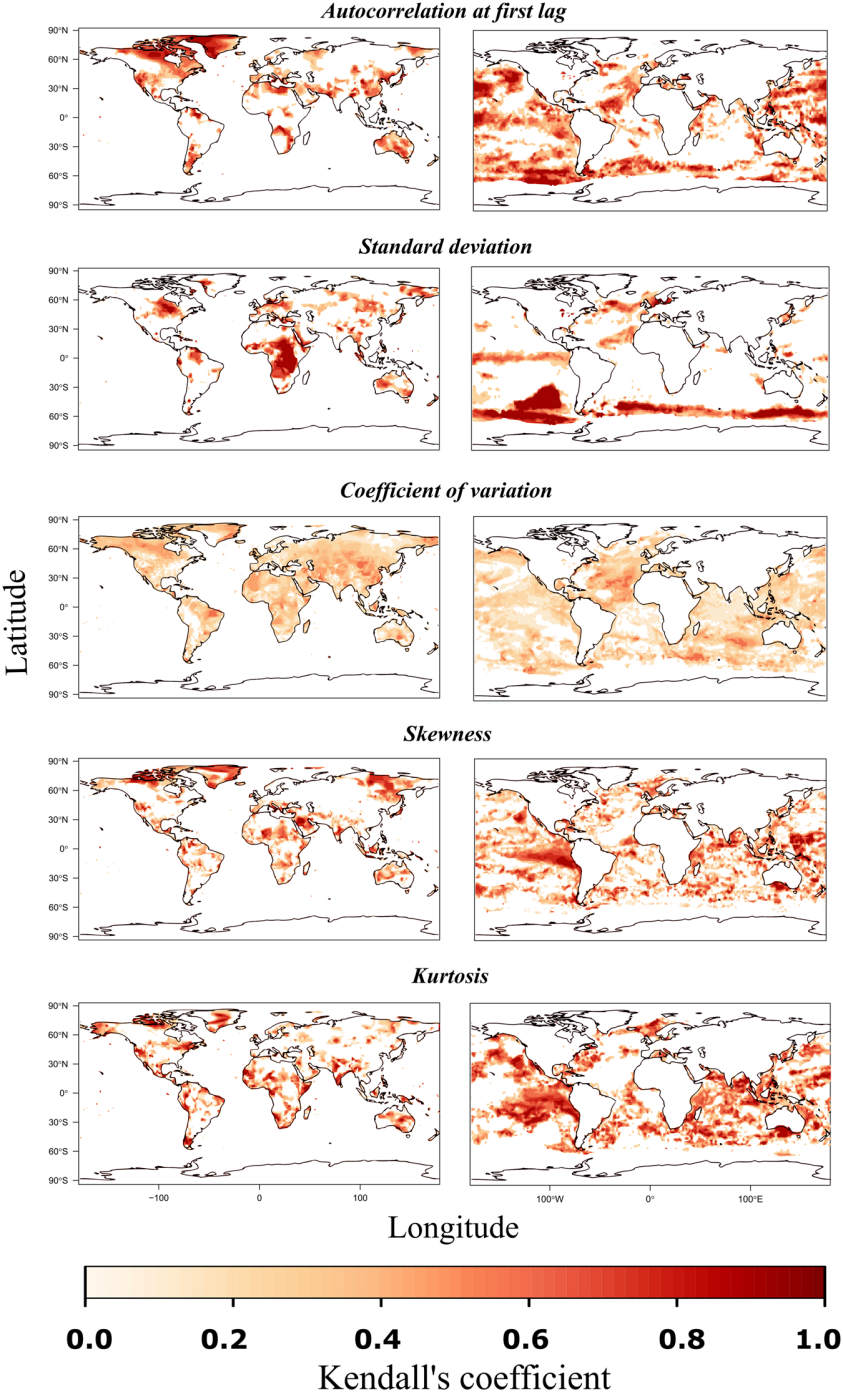
Temporal trends (Kendall’s τ) in five EWI in terrestrial (left column) and marine (right column) systems. Since negative trends are not indicative of approaching transitions, only positive trends are represented for graphical purposes.

For all EWIs, we found large spatial variations in their temporal trends but the patterns highlighted greatly varied with respect to their geographical distribution and the strength of their trends (Fig. 1). For instance, Greenland and Northeast Canada displayed strong and positive trends for the autocorrelation at first lag, but no trends for the standard deviation. A similar discrepancy between these two indicators was found around the Equator in Africa, but in the opposite direction. In the marine system, we found a strong signal in the south Pacific with respect to standard deviation and autocorrelation at first lag but not signal for skewness and kurtosis (Fig. 1).

**Figure 2:**
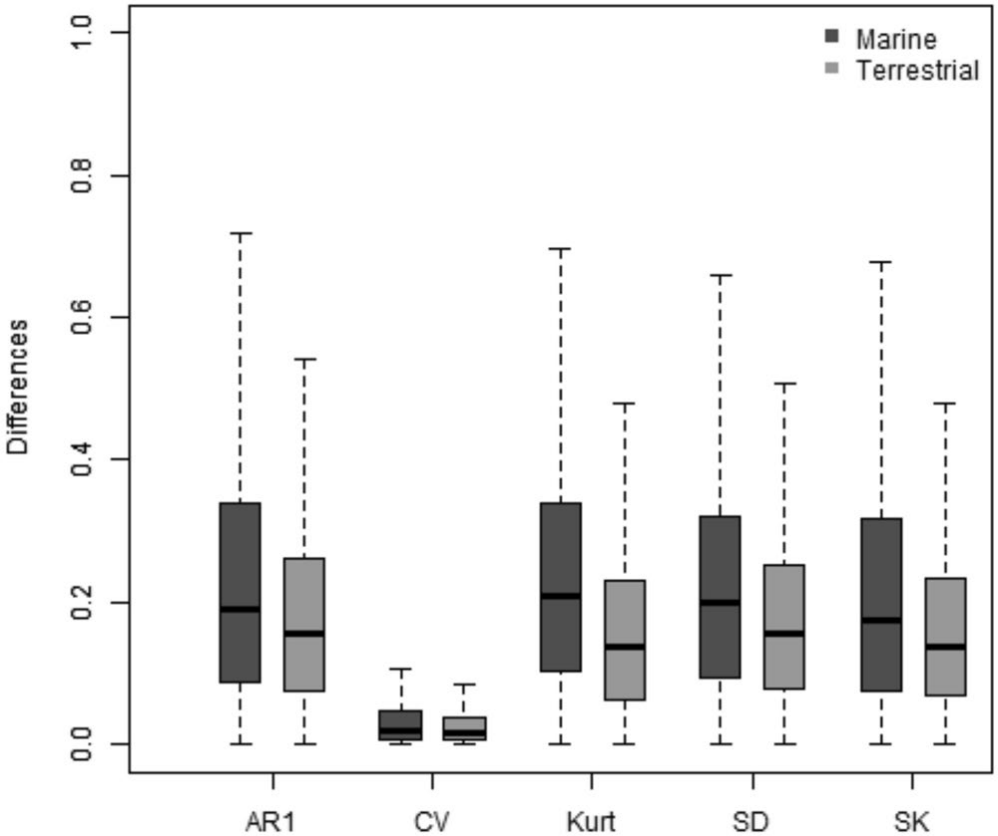
Contribution of the five EWIs to the trends estimated by the composite metric. The contribution was calculated as the absolute difference between the original trends and the trends obtained after one of the five EWIs had been removed for the construction of the composite metric. The box represent the lower quartile, median, and upper quartile of the data while whiskers point to minimum and maximum values. AR1 = autocorrelation at first lag; CV = coefficient of variation; Kurt = kurtosis; SD = standard deviation and SK = skewness.

**Figure 3:**
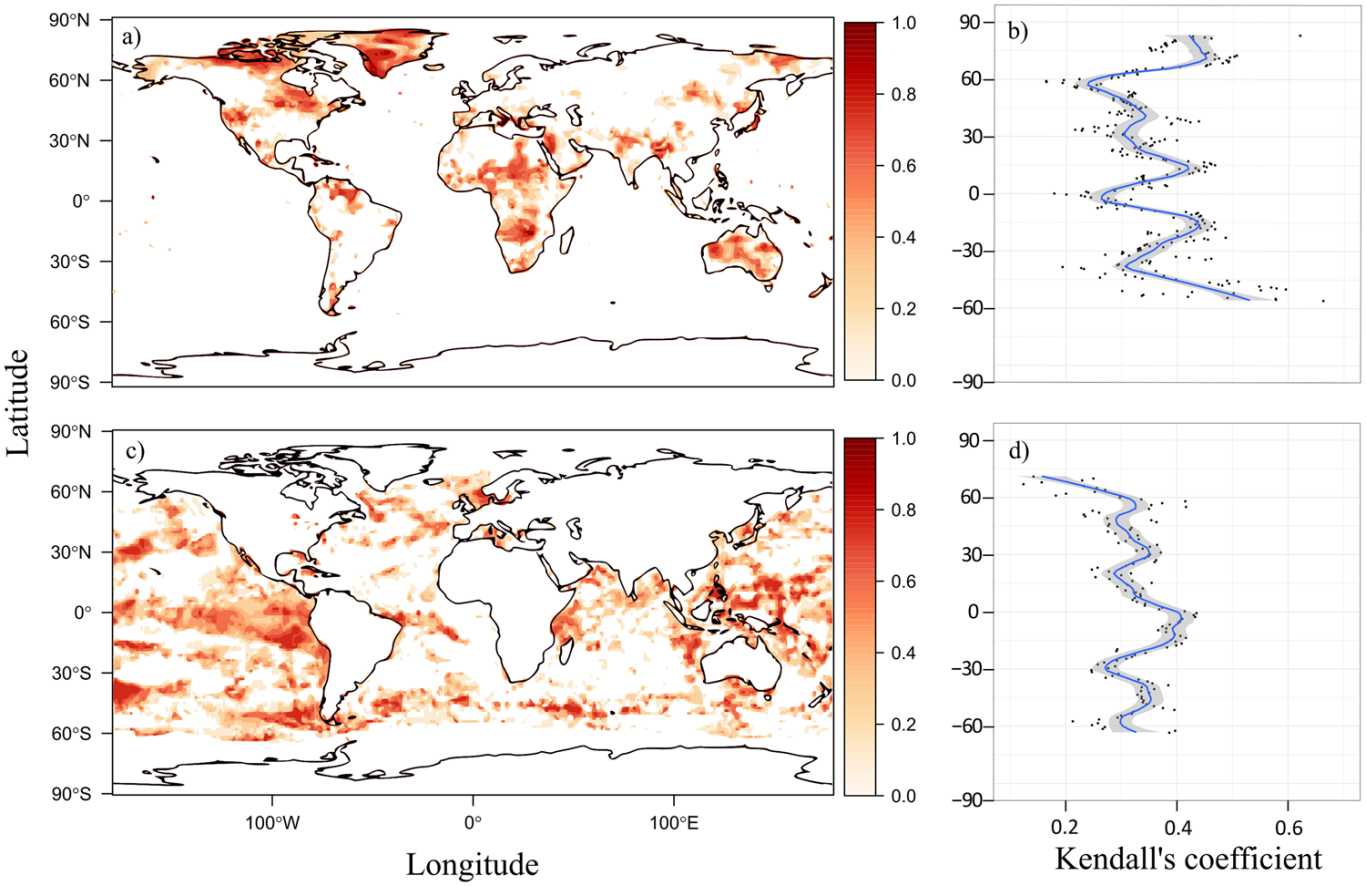
Worldwide trends (Kendall’s τ) and latitudinal gradient in the composite EWI in terrestrial (**a**,**b**) and marine (**c**,**d**) systems. Since negative trends are not indicative of approaching critical transitions, only positive trends are represented for graphical purposes (high resolution maps are provided in the supplementary material for both terrestrial and marine systems; Figs S6 and S7, respectively). Latitudinal gradient is represented as the average value of positive trends calculated at each latitudinal band. To help visualize the gradient we overlaid a smooth linear component. This component was estimated with a generalized linear additive model with average Kendall’s τ as the dependent variable and Latitude as the independent variable. The degree of smoothness was estimated within the model using penalized likelihood maximization.

Among the five EWIs, four contributed approximately equally to the trends estimated with the composite EWI whereas the coefficient of variation had less influence (Fig. 2). Trends for the composite EWI were also greatly variable in space, pointing to areas with strong and positive trends where the congruency among individual EWIs is maximized in both terrestrial (e.g. Center of Australia, Greenland; Fig. 3a) and marine (e.g. the equatorial Pacific; Fig. 3c) systems. The congruency among EWIs was further reinforced by the fact that more than 90% of the pixels showing positive trends for the composite EWI in terrestrial (94.5%) and marine (90.2%) systems also showed positive trends for at least three of the five EWIs. Using a type I error rate of 10% (see methods), we found that pixels showing the strongest trends (i.e. above 0.5) were mostly true positives (Supplementary Material Fig. S1) and represented 27% and 22% of the pixels displaying positive trends in terrestrial and marine systems, respectively.

In both systems, the average value of pixels with positive trends varied with latitude, although in different ways. Marine systems exhibited a strong signal around the Equator, which tended to decrease at higher or lower latitudes with a particularly low signal at the northern extreme of the latitudinal gradient (Fig. 3d). The pattern was different in terrestrial systems. The signal was low around the equator and the two Tropics (i.e. 30° North and 30° South) but was higher in between those latitudes (Fig. 3b). We further found a high signal at both extreme of the latitudinal gradient. Results obtained for the composite EWI were overall robust to varying window length and detrending bandwidth, with only slight variations in the estimated trends (Supplementary Material Figs S2–S4). Given that skewness and kurtosis have mixed support in the literature, we also investigated spatial patterns in trends using a composite metric not including them. Overall, we found no obvious qualitative differences relative to the patterns highlighted with the full composite metric, although trends in some areas (e.g. Southern part of the latitudinal gradient in marine systems) were quantitatively affected (Supplementary Material Fig. S5).

**Figure 4:**
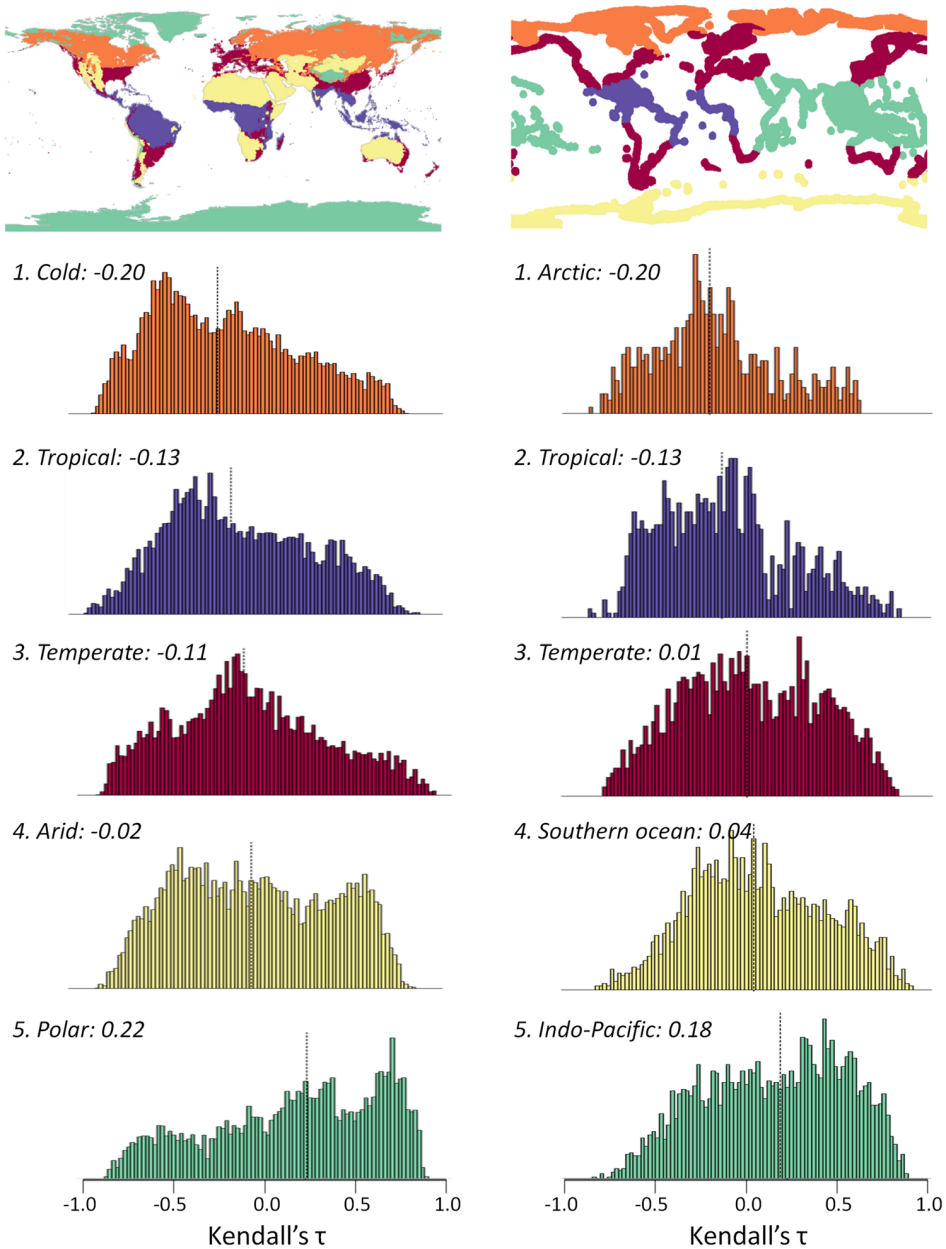
Map of biomes and histograms of the trends within each biome for terrestrial (left column) and marine (right column) systems. Histograms are ordered by increasing trends according to the median value of the composite EWI (black dotted line; values are shown next to the name of each ecoregion). Some ecoregions were grouped together to improve graphical representation, but detailed information for individual ecoregions is provided in the supplementary material (Tables S2 and S3). The delineation of biomes in terrestrial and marine systems was obtained from the Köppen-Geiger climatic classification (http://koeppen-geiger.vu-wien.ac.at) and the World Wildlife Fund (WWF) Marine Ecoregions Of the World.

Within terrestrial systems, polar ecoregions showed the strongest trends in the composite EWI whereas cold and tropical regions were the ecoregions showing the lowest trends (Fig. 4). At a finer scale, Mediterranean forests, woodlands and scrub showed the strongest trends (Supplementary Material Table S2). We also found differences within marine systems. The Indo-Pacific and the Southern Ocean ecoregions exhibited the strongest trends in the composite EWI, whereas the Arctic ecoregion showed the lowest trends (Fig. 4). Four marine ecoregions (temperate South-America, tropical Eastern-Pacific, Western Indo-Pacific and temperate Australasia) displayed particularly strong trends (Supplementary Table S3).

## Discussion

The use of EWIs as tools to identify critical transition has gained substantial interest in the field of ecology over recent years^6^. Most studies conducted so far used experiments^37^, simulations^10^, or empirical data from systems in which critical transitions occurred in the past^17^ to investigate the ability of EWIs to detect tipping points before they occur. These retrospective studies revealed that several statistical features of time series can be used as EWIs of regime shifts, thus providing a framework to use them prospectively in order to explore systems in which tipping points are likely to be reached in the future. For instance, temperature has undergone several transitions in the past between a cold and a warm state^17^ and may do so in the near future^38^. Here, we aimed to generate “risk evaluation maps” of upcoming tipping points in the temperature regime in both terrestrial and marine systems. Although the underlying mechanisms cannot be assessed with certainty, such exploration is of interest from both a conservation and a management point of view because ecological systems are expected to track changes in the climate system.

Overall, we found strong and positive long-term trends in several EWIs, suggesting that some areas over the world may undergo a transition toward a qualitatively different climate state in the future. Regardless of the EWI considered, trends were not uniformly distributed across the world which can be explained by several factors. For instance, if we consider that shifts in temperature time series occur for the same value of the forcing regime (e.g. land-use changes), then spatial variations in that regime will generate spatial variations in EWIs. Alternatively, transitions between alternative stable states may occur for different values of the forcing regime^39^ and may also explain spatial variations in EWIs. Temperature is also influenced by several components (e.g. surface reflectivity due to land-use changes, changes in greenhouse gases concentration) of which each can display a nonlinear response to some variables on different time scales^40^. Beyond spatial variations, we found differences regarding the information provided by the different EWIs either in their spatial distribution or in the strength and the sign of their trends. Those differences could be due to differences in the underlying phenomenon driving the system toward a tipping point. For instance, areas showing positive trends for the coefficient of variation, skewness and kurtosis (e.g. Amazonian forest) could indicate flickering where the system switches back and forth between alternative stable states in response to externally imposed perturbations having large impacts^20^. In contrast, positive trends for autocorrelation and variance (e.g. south Pacific) could suggest that CSD is the underlying phenomenon^41^ and therefore that small and gradual (as opposed to large and stochastic) changes in external conditions are driving the system to the bifurcation point^42^. More complex combinations of trends can occur depending on the behavior and the mathematical properties of the systems. For instance, when the rates of change of the state variable are slow relative to the frequency characteristics of the regime, the variance may decrease close to a tipping point while the autocorrelation may increase^24^.

The trends highlighted for the five EWIs can also involve mechanisms that are not related to critical transitions^43,44^. For example, changes in noise and certain external drivers can push a system from a point attractor to a non-point attractor, where it starts to fluctuate more frequently^45^. Likewise, systems that have experienced strong environmental perturbations (e.g. hurricanes) can display transient responses before returning to their equilibrium^46^. These changes do not involve tipping points or shifts between alternative stable states but would still be characterized by an increase in the variability of the system, mimicking an approaching critical transition, even though temporal autocorrelation is not expected to change in those cases^41^. The direct consequence of this is that the use of a single EWI can be flawed and lead to false alarms under certain conditions^22,23^. To mitigate limitations and sources of uncertainty associated with the use of single EWI, several authors e.g.^10,16,18^ have suggested to use multiple EWIs, while others have argued that the detection of tipping points can only be achieved by considering both variance and autocorrelation^47^. Since multiple EWIs have been shown to increase at the vicinity of tipping points, either due to flickering or CSD^20^, the use of a composite indicator might be particularly useful because it would make it possible to highlight congruent patterns among various EWIs, and thus provide further support for upcoming critical transitions^8,27^.

Using such an indicator, we found that particular areas over the world showed strong and positive trends with about one fourth of pixels in both systems being true positives. From those pixels at least, it suggest that some areas (e.g. Australia, Greenland, Equatorial Pacific) are likely to experience a drastic shift in the temperature regime in the future. For the remaining pixels (i.e. those showing negative trends or positive but weak trends), although they might indicate that the system is not approaching a critical transition, they might as likely be false negatives, given that EWIs may sometimes fail to indicate a critical transition^25,48^. Regarding our composite indicator, false negatives can occur in regions where EWIs are expected to have opposite signs, such as when different mechanisms are at play. Those cases are not easy to distinguish without additional knowledge about the behavior and the mathematical properties of the system. When such a knowledge is accessible, one can refine the composite indicator to reflect congruent trends among EWIs. For instance, ref.^27^ multiplied the trends of some of their EWIs by −1 to match the positive trend expected for other EWIs. The spatial scale and the number of time series considered here do not make it possible to use such an approach and we therefore cannot rule out the possibility that the absence of signal found in some areas (e.g. Boreal forest ecoregions which have undergone strong changes in land-cover across the past decades) are false negatives.

As already shown for other facets of climate change^3,49^, we found that the trends obtained from the composite EWI varied with latitude and between ecoregions for both terrestrial and marine systems. In particular, we found that both extremes of the latitudinal gradients along with areas located in between the Equator and the Tropics displayed strong signal in terrestrial systems, whereas the signal was the strongest around the Equator in marine systems. This discrepancy between the two systems might either stem from differences in the forcing regime or in the physical climate-related mechanisms between the two systems. For instance, deepening of the mixed layer is a physical mechanism related to water depth and wind speed that can explain the critical slowing down of sea surface temperatures in the North Pacific domain^50^. By contrast, positive feedbacks related to changes in snow cover can influence surface reflectivity (albedo) which can amplify changes at the poles^51^ and explain the strong signals found in the northern part of the latitudinal gradient in terrestrial systems. The contrasting pattern (i.e. low signal) found at this latitude for the marine system could, on the other hand, be explained by the fact that this region is already beyond a bifurcation point and in the middle of a critical transition to a warmer state.

Importantly, most of the areas showing the strongest signals in the terrestrial system correspond to areas that have undergone strong changes in land-use (e.g. Australia, west coast of the US)^52^ or land-cover (e.g. forest cover in Zambia, Angola, Surinam; snow cover in north Canada)^53^ over the past decades. By modifying the energy budget balance, these changes have been shown to have consequences on several climatic variables, including temperature^35,36^, and our results suggest that these changes might trigger drastic changes in the temperature regime in the future. The strong signal highlighted in the northern part of the Canada is particularly concerning as a large proportion of this area is composed of permafrost containing large amounts of carbon with a potential high incidence for global warming^54^. Likewise, the strong signal found in the Equatorial Pacific Ocean where the El Niño-Southern Oscillation phenomenon (ENSO) takes place is worrisome given that this phenomenon is one of the most important process underlying climate variability at large spatial scales^55^ and that year to year transitions between a warm (El-Niño) and a cold (La Niña) state have already been highlighted^56^. Overall, it is interesting to note that the areas showing the strongest signals (e.g. ENSO, Greenland) closely match with the tipping elements hypothesized by ref.^57^ with respect to critical transitions in the climate system.

Within systems, we found strong variations between ecoregions, as already highlighted, for climate velocities^3,58^ or other components of the climate system^59^. This suggests that the probability of upcoming critical transitions is likely to vary depending on the ecological conditions prevailing in a given region and especially the changes to which this region has been submitted to. For instance, Mediterranean ecoregions have undergone large changes in land-use over the last decades^60^ and were those showing the strongest evidence of upcoming critical transitions. Likewise, some ecoregions rated as “arid” (e.g. Australia) showed strong signals, a pattern that is in line with some previous research e.g. ref.^61^ showing that severe overgrazing and resultant land degradation led to higher temperatures in these ecoregions. Given that these ecoregions host many endemic species^62^, our results warn against future changes regarding species extinction risk, rearrangement of ecological communities, and ecosystem functioning.

## Conclusion

The recognition that human activities have already altered the climate system to some extent and that further human-forced changes in the Earth’s climate system seems inevitable^33^ has led ecologists to reduce uncertainty and improve accuracy in model projections in order to better forecast the impact of human activities on biodiversity and ecosystems^4^. The development of EWIs of regime shifts represents a promising avenue in this context because it makes it possible to detect tipping points in dynamic systems before they occur and raise alarms to prevent shifting from one state to another^6^. Differences in the inferences obtained from different EWIs, although expected to behave in the same way at the vicinity of tipping point, has questioned the use of single EWI to detect upcoming critical transitions^47^. We have shown that using a composite indicator may represent an interesting alternative by making it possible to unravel congruent trends among individual EWIs and identify areas or systems where the likelihood of approaching critical transition is important. Nonetheless, the results provided here are insufficient in the sense that they indicate that “something” important may be about to happen in some areas but do not tell us what precisely that “something” would be, when exactly it will happen and what is the underlying mechanism. Consequently, the areas highlighted here as showing a strong probability of upcoming critical transitions warrants further investigation. Indeed, although it is likely that land-use and land-cover changes might be involved in the trends highlighted here, more knowledge is needed to precisely identify what are the underlying processes. Repeating the above analyses at a smaller spatial scale and with datasets having higher spatio-temporal resolutions, collected from governmental agencies or local institutions, for both biotic and/or abiotic variables might help in this regard. In particular, it would help (i) clarify what are the processes pushing the climate system toward a tipping point, (ii) evaluate the time before the critical transition, and (iii) quantify the potential consequences for biodiversity and ecosystem functioning.

## Methods

### Datasets

We sourced monthly time series from 1950 to 2014 from the Center Research Unit (CRU V3.23; https://crudata.uea.ac.uk/cru/data/hrg/) at a 0.5° resolution for air temperature (AT) and from the Met Office Hadley Center (HadISST; http://www.metoffice.gov.uk/hadobs/hadisst/) at a 1° resolution for sea surface temperature (SST). These raster maps are composed of 67325 and 32619 monthly observations time series for AT and SST, respectively. The number reported for the latter excludes pixels that were mostly composed of ice as those pixels are not temperature time series and can’t be analyzed with EWIs.

To determine whether some ecoregions were more likely to approach a critical transition than others, as already shown for other facets of change^3,4^, we extracted terrestrial biomes from the Köppen-Geiger climatic classification (http://koeppen-geiger.vu-wien.ac.at) whereas for sea surface temperature we compiled biomes from the World Wildlife Fund (WWF) Marine Ecoregions Of the World (MEOW; http://www.worldwildlife.org/). The year band used for the Köppen-Geiger classification was 1986–2010. Note that we haven’t used the WWF classification for the terrestrial system because ecoregions were not comparable to the marine ecoregions. We nonetheless report result for this classification in the supplementary material (Table S2).

### Simple and composite EWI

Temperature time series were analyzed separately after removing the seasonal component. For this purpose, we decomposed the times series into seasonal, trend, and irregular components using a moving average procedure^63^ and only conserved the last two components for the remaining analyses. We then filtered out long-term trends in the time series to achieve stationarity using a Gaussian kernel smoother with a fixed bandwidth of 50^17^. We chose a bandwidth of 50 because preliminary analyses performed on several pixels revealed that this value was appropriate. From the resulting time series, we compiled eight EWIs using a sliding window of half the length of the original time series using the package earlywarnings^64^ within the R environment software^65^. However, we did not conserve all EWIs as some of them were highly correlated (ρ > 0.99; p < 0.001) and thus redundant (Supplementary Material Table S1). Consequently, our study is based on five EWIs: the first order autoregressive coefficient (i.e. a measure of temporal autocorrelation at first lag), the standard deviation, the coefficient of variation, the skewness, and the kurtosis. Although the first three EWIs are all expected to increase when approaching a critical transition, trends in skewness and kurtosis depend on whether the transition is toward an alternative state that is larger or smaller than the present state^10^. Nonetheless, given the global warming context, changes in the temperature regime, if any, are likely to be toward a warmer state and we therefore expect a rise in both indicators^22,41^. This is because an increase in the frequency of extreme warm temperatures is expected to create an asymmetry towards the right hand of the distribution while, at the same time, the distribution is expected to become more “leptokurtic” with an increase in the frequency of rare values.

From the five above-mentioned EWIs, we computed a composite metric to determine whether it can reinforce evidence of an approaching transition relative to individual EWI. As a first step, we normalized each of the five EWIs by subtracting the long-run average of that indicator from the value of that indicator at time t, and divided it by the long-run standard deviation (i.e. z-score transformation)^8^. To obtain the composite EWI, we then summed the normalized values of individual EWIs at each time step for each pixel, providing us time series for the composite EWI. For all EWIs (individuals and composite), we used Kendall’s τ rank correlation coefficient as a measure of their tendency to increase or decrease over time in each pixel, as classically done and recommended^17^. From the trends obtained, we generated “risk evaluation maps” for each EWI using the packages rworldmap^66^ and rasterVis^67^ to highlight spatial variations in the trends obtained for the different EWIs. We do not report negative trends because they are non-informative of approaching a critical transition and might rather indicate “critical speeding-up” whereby resilience to perturbations increases after the system has crossed a tipping point^68^. To evaluate the contribution of the five EWIs to the trends estimated with the composite EWI, we used a cross-validation approach where we (i) recalculated the composite EWI by removing one of the five EWIs, (ii) re-estimated the trend, and (iii) calculated the absolute difference between the new trend and the original trend. To determine how trends in the composite indicator varied with latitude, we focused on positive trends (i.e. positive Kendall’s τ coefficient) and calculated the average value of trends for each latitudinal band. Given that skewness and kurtosis have mixed support from the theoretical literature^41^, we also generated “risk evaluation maps” using a composite metric computed without these two EWIs (see Supplementary Material Fig. S5).

Because trends in EWIs can be sensitive to the choice of sliding window and detrending bandwidth^17^, we repeated our analyses for different combinations of these parameters. We calculated the coefficient of variation of the trends obtained from the different combinations and used this as a measure of sensitivity of our results to the choice of sliding window and detrending bandwidth. A high value indicated a strong influence of these parameters on the estimated trend and thus a low robustness of our results.

### Identifying false positives

For EWIs to be ecologically meaningful, one has to test whether the observed trends are different from the ones that would have been obtained by chance. To test whether trends computed for the composite EWI in each pixel were true positives, we generated 1,000 surrogate time series with the same length and autocorrelation structure as the original residual time series i.e. after the seasonal component and the long-term trend have been removed^41^. The autocorrelation structure of the surrogate time series was set to the value estimated from the best linear autoregressive moving average model (ARMA) fitted to the original time series and identified using a stepwise selection procedure based on AIC. For each surrogate time series we then computed the composite EWI and calculated its long-term trend, as explained above. As a result we obtained a distribution of Kendall’s τ coefficients under the null hypothesis that there is no temporal trend in the composite EWI. Significance was defined as the percentage of simulated trends showing higher values than the observed trend with a threshold fixed to 10%. Because the overall procedure was very time-consuming, we only tested the significance of trends for the composite EWI.

### Data availability statement

All the data used in this manuscript are freely available on the web at the following addresses: - https://crudata.uea.ac.uk/cru/data/hrg/. - http://www.metoffice.gov.uk/hadobs/hadisst/. - http://koeppen-geiger.vu-wien.ac.at/. - http://www.worldwildlife.org/

## Electronic supplementary material

Supplementary information

## References

[1] Beaumont LJ, Pitman A, Perkins S, Zimmermann NE, Yoccoz NG (2011). Impacts of climate change on the world’s most exceptional ecoregions. Proc. Natl. Acad. Sci. USA.

[2] Pacifici, M., Visconti, P. & Rondinini, C. A framework for the identification of hotspots of climate change risk for mammals. *Glob. Chang. Biol*. 10.1111/gcb.13942 (2017). 10.1111/gcb.13942 29031011

[3] Loarie SR (2009). The velocity of climate change. Nature.

[4] Garcia RA, Cabeza M, Rahbek C, Araújo MB (2014). Multiple dimensions of climate change and their implications for biodiversity. Science.

[5] Scheffer M, Carpenter S, Foley Ja, Folke C, Walker B (2001). Catastrophic shifts in ecosystems. Nature.

[6] Scheffer M (2009). Early-warning signals for critical transitions. Nature.

[7] Vasilakopoulos P, Marshall CT (2015). Resilience and tipping points of an exploited fish population over six decades. Glob. Chang. Biol..

[8] Drake JM, Griffen BD (2010). Early warning signals of extinction in deteriorating environments. Nature.

[9] Beaugrand G (2015). Theoretical basis for predicting climate-induced abrupt shifts in the oceans. Philos. Trans. B.

[10] Guttal V, Jayaprakash C (2008). Changing skewness: An early warning signal of regime shifts in ecosystems. Ecol. Lett..

[11] Carpenter, S. R., Kinne, O. & Wieser, W. *Regime shifts in lake ecosystems: pattern and variation*. **15**, (International Ecology Institute Oldendorf/Luhe 2003).

[12] Jackson JB (2001). Historical overfishing and the recent collapse of coastal ecosystems. Science.

[13] Gunderson, L. H. & Pritchard, L. *Resilience and the behavior of large-scale systems*. **60**, (Island Press, 2012).

[14] Clements CF, Drake JM, Jason IG, Ozgul A (2015). Factors Influencing the Detectability of Early Warning Signals of Population Collapse. Am. Nat..

[15] Mäler K-G (2000). Development, ecological resources and their management: A study of complex dynamic systems. Eur. Econ. Rev..

[16] Lenton TM (2011). Early warning of climate tipping points. Nat. Clim. Chang..

[17] Dakos V (2008). Slowing down as an early warning signal for abrupt climate change. Proc. Natl. Acad. Sci. USA.

[18] Lenton TM, Livina VN, Dakos V, van Nes EH, Scheffer M (2012). Early warning of climate tipping points from critical slowing down: comparing methods to improve robustness. Philos. Trans. R. Soc. A Math. Phys. Eng. Sci..

[19] Dakos V, Carpenter SR, van Nes EH, Scheffer M (2014). Resilience indicators: prospects and limitations for early warnings of regime shifts. Philos. Trans. R. Soc. B Biol. Sci..

[20] Dakos V, van Nes EH, Scheffer M (2013). Flickering as an early warning signal. Theor. Ecol..

[21] Wang R (2012). Flickering gives early warning signals of a critical transition to a eutrophic lake state. Nature.

[22] Perretti CT, Munch SB (2012). Regime shift indicators fail under noise levels commonly observed in ecological systems. Ecol. Appl..

[23] Kéfi S, Dakos V, Scheffer M, Van Nes EH, Rietkerk M (2013). Early warning signals also precede non-catastrophic transitions. Oikos.

[24] Dakos V, Van Nes EH, D’Odorico P, Scheffer M (2012). Robustness of variance and autocorrelation as indicators of critical slowing down. Ecology.

[25] Hastings A, Wysham DB (2010). Regime shifts in ecological systems can occur with no warning. Ecol. Lett..

[26] Gsell, A. S. *et al*. Evaluating early-warning indicators of critical transitions in natural aquatic ecosystems. *Proc. Natl. Acad. Sci*. 201608242 10.1073/pnas.1608242113 (2016). 10.1073/pnas.1608242113 PMC5167183 27911776

[27] Clements CF, Ozgul A (2016). Including trait-based early warning signals helps predict population collapse. Nat. Commun..

[28] Biggs R, Carpenter SR, Brock WA (2009). Turning back from the brink: Detecting an impending regime shift in time to avert it. Proc. Natl. Acad. Sci..

[29] Takimoto G (2009). Early warning signals of demographic regime shifts in invading populations. Popul. Ecol..

[30] Zeng N, Neelin JD, Lau KM, Tucker CJ (1999). Enhancement of interdecadal climate variability in the Sahel by vegetation interaction. Science (80-.)..

[31] van Nes EH (2015). Causal feedbacks in climate change. Nat. Clim. Chang..

[32] Alley RB (2005). AbruptClimate Change. Science (80-.)..

[33] IPCC. Climate Change 2014: Synthesis Report. Contribution of Working Groups, 1, 11 and 111 to the Fifth Assessment Report of the Intergovernmental Panel on Climate Change [Core Writing Team, R.K. Pachauri and L. A. Myers (eds)]. 10.1017/CBO9781107415324 (2014).

[34] Kalnay E, Ming C (2003). Impact of urbanization and land-use change on climate. Nature.

[35] Bright RM (2017). Local temperature response to land cover and management change driven by non-radiative processes. Nat. Clim. Chang..

[36] Alkama R, Cescatti A (2016). Biophysical climate impacts of recent changes in global forest cover. Science (80-.)..

[37] Carpenter SR, Ludwig D, Brock WA (1999). Management of Eutrophication for Lakes Subject to Potentially Irreversible Change. Ecol. Appl..

[38] Barnosky AD (2012). Approaching a state shift in Earth/’s biosphere. Nature.

[39] van Nes EH, Scheffer M (2005). Implications of Spatial Heterogeneity for Catastrophic Regime Shifts in Ecosystems. Ecology.

[40] Rypdal, M. Early-Warning Signals for the onsets of Greenland Interstadials and the Younger Dryas-Preboreal transition. *J. Clim*. JCLI-D-15-0828.1 10.1175/JCLI-D-15-0828.1 (2016).

[41] Dakos, V. *et al*. Methods for detecting early warnings of critical transitions in time series illustrated using simulated ecological data. *PLoS One* **7** (2012). 10.1371/journal.pone.0041010 PMC3398887 22815897

[42] Lenton TM (2012). What early warning systems are there for environmental shocks?. Environ. Sci. Policy.

[43] Hughes TP, Carpenter S, Rockström J, Scheffer M, Walker B (2013). Multiscale regime shifts and planetary boundaries. Trends Ecol. Evol..

[44] Hughes TP, Linares C, Dakos V, van de Leemput IA, van Nes EH (2013). Living dangerously on borrowed time during slow, unrecognized regime shifts. Trends Ecol. Evol..

[45] Ives AR, Carpenter SR (2007). Stability and diversity of ecosystems. Science (80-.)..

[46] Hastings A (2001). Transient dynamics and persistence of ecological systems. Ecol. Lett..

[47] Ditlevsen PD, Johnsen SJ (2010). Tipping points: Early warning and wishful thinking. Geophys. Res. Lett..

[48] Burthe SJ (2016). Do early warning indicators consistently predict nonlinear change in long-term ecological data?. J. Appl. Ecol..

[49] Burrows MT (2011). The Pace of Shifting Climate in Marine and Terrestrial Ecosystems. Science (80-.)..

[50] Boulton CA, Lenton TM (2015). Slowing down of North Pacific climate variability and its implications for abrupt ecosystem change. Proc. Natl. Acad. Sci. USA.

[51] Holland MM, Bitz CM (2003). Polar amplification of climate change in coupled models. Clim. Dyn..

[52] Foley JA (2005). Global consequences of land use. Science (80-.)..

[53] Hansen MCC (2013). High-Resolution Global Maps of 21st-Century Forest Cover Change. Science (80-.)..

[54] Lenton TM (2012). Arctic climate tipping points. Ambio.

[55] Power S, Delage F, Chung C, Kociuba G, Keay K (2013). Robust twenty-first-century projections of El Niño and related precipitation variability. Nature.

[56] Ludescher, J. *et al*. Very early warning of next El Niño. *Proc. Natl. Acad. Sci*. **111** (2014). 10.1073/pnas.1323058111 PMC3926055 24516172

[57] Lenton TM (2008). Tipping elements in the Earth’s climate system. Proc. Natl. Acad. Sci..

[58] García Molinos J (2015). Climate velocity and the future global redistribution of marine biodiversity. Nat. Clim. Chang..

[59] Ordonez, A., Williams, J. W. & Svenning, J. Mapping climatic mechanism likely to favour the emergence of novel communities. *Nat. Clim. Chang*. **6** (2016).

[60] Serra P, Pons X, Saurí D (2008). Land-cover and land-use change in a Mediterranean landscape: A spatial analysis of driving forces integrating biophysical and human factors. Appl. Geogr..

[61] Balling RC (1998). Impacts of land degradation on historical temperature records from the Sonoran Desert. Clim. Change.

[62] Myers N, Mittermeier RA, Mittermeier CG, da Fonseca GAB, Kent J (2000). Biodiversity hotspots for conservation priorities. Nature.

[63] Kendall, M. G. & Stuart, A. The advanced theory of statistics, vol. III. *Hafner New York* (1976).

[64] Dakos, V. & Lahti, L. R Early Warning Signals Toolbox. *R Proj. Stat. Comput*. (2013).

[65] R Core Team. *R: A language and environment for statistical computing*. (URL http://www.R-project.org/ 2014).

[66] South A (2011). rworldmap: A New R package for Mapping GlobalData. The R Journal.

[67] Perpiñán, O. & Hijmans, R. rasterVis (2016).

[68] Pananos AD (2017). Critical dynamics in population vaccinating behavior. Proc. Natl. Acad. Sci..

