# Supplementary material for: Global assessment of early warning signs that temperature could undergo regime shifts
Source: Sci Rep. 2018 Jul 3;8:10058. doi: 10.1038/s41598-018-28386-x (PMC6030089; doi:10.1038/s41598-018-28386-x)

Supporting Information - *Global assessment of early warning signs that temperature could undergo regime shifts*

Mathieu Chevalier* & Gaël Grenouillet

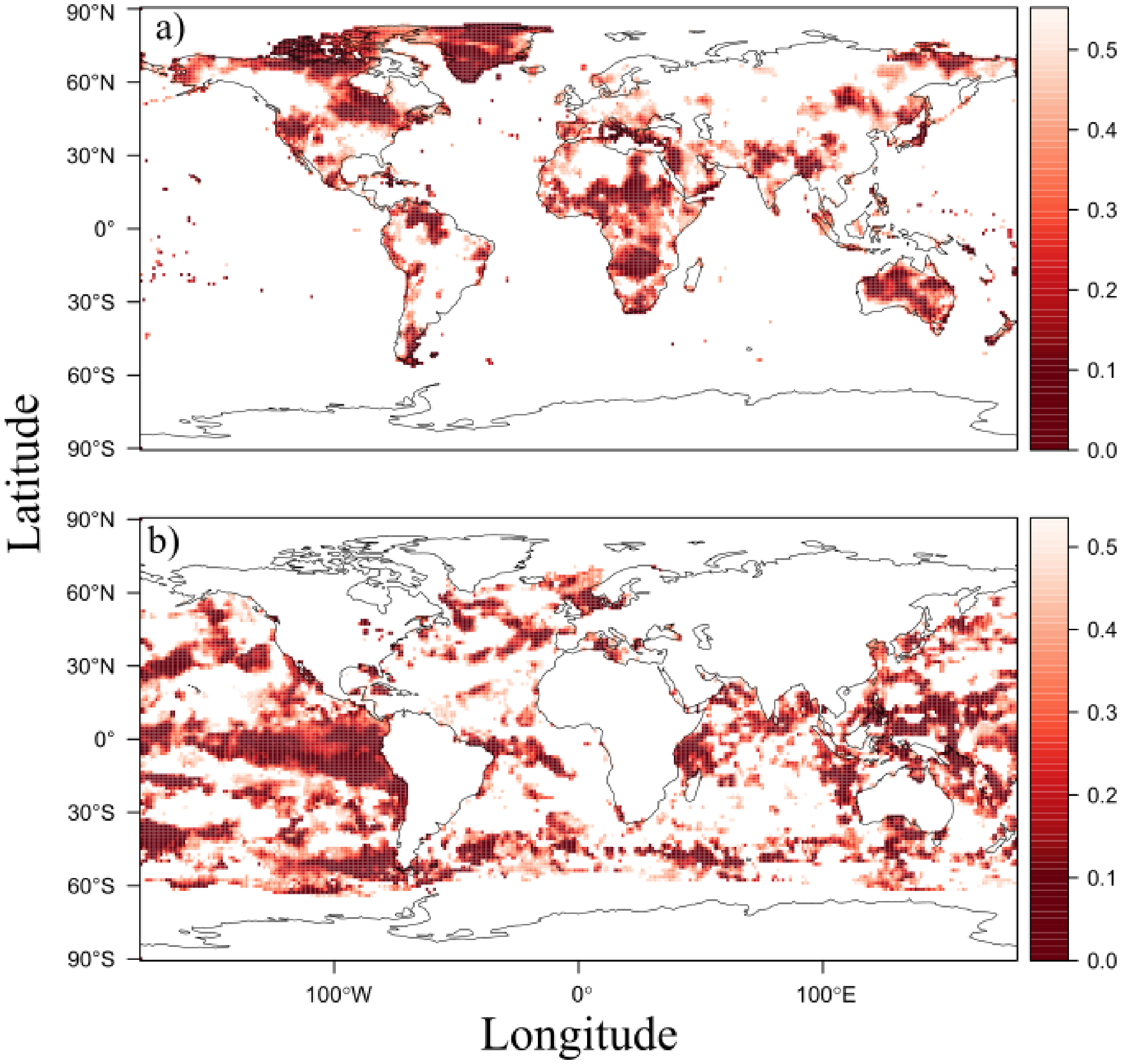

Figure S1. Spatial representation of the uncertainty of the trends computed from the composite EWI in terrestrial (a) and marine (b) systems. The scale represent the proportion of trends obtained from 1,000 surrogate time series presenting values (i.e. Kendall's coefficients) that are equal or superior to the observed trend. The darker the colours, the lower the proportion and therefore the higher the probability that the observed trend is a true positive. The surrogate time series were simulated with the same correlation structure and probability distribution as the original time series but assuming a linear stationary process.

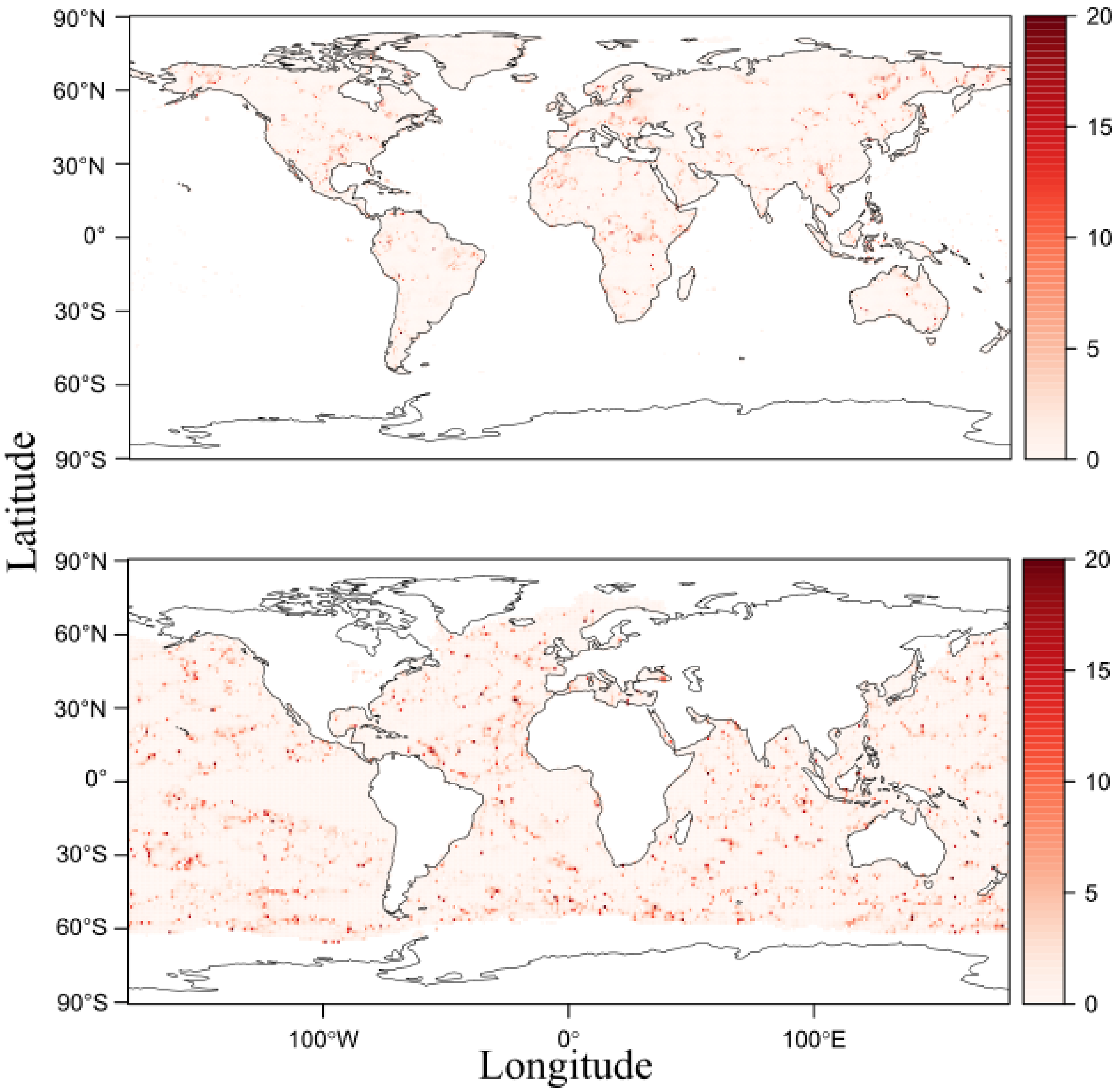

Figure S2. Global maps of the coefficient of variation of temporal trends (in absolute values) in the composite indicator with different values of window length (varying from 40 to 60% of the length of the original time series, by steps of 5%) and detrending bandwidth (varying from 40 to 60 by steps of 5). Red colors indicate a high coefficient of variation and thus a low congruence between the trends estimated with different parameter values (i.e. low robustness of our result).

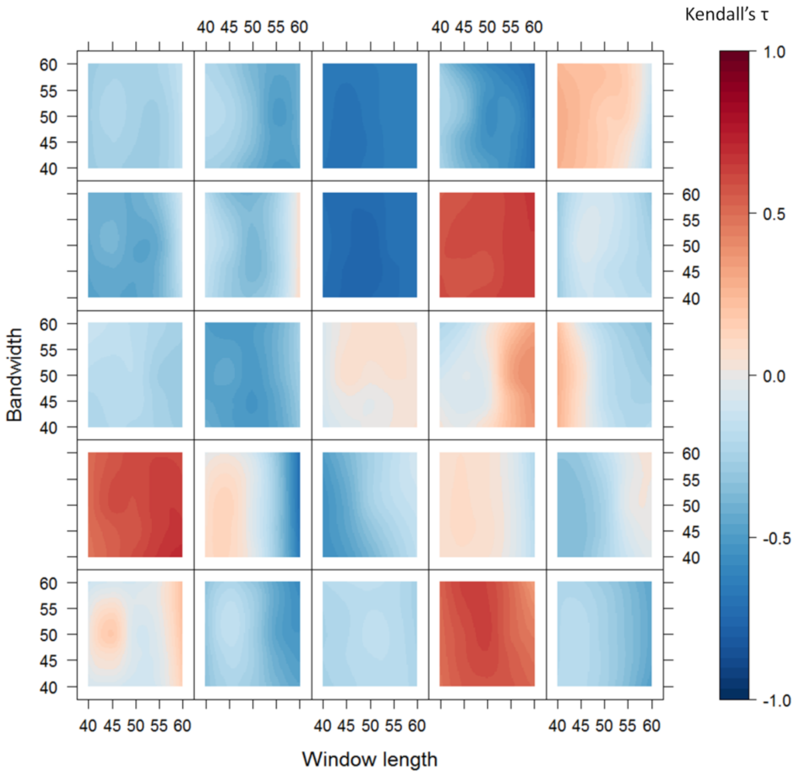

Figure S3. Sensitivity analysis (contourplots) of the temporal trends in the composite EWI represented by the estimated values of Kendall's τ coefficient for a variety of window length and detrending bandwidth for 25 cells randomly chosen among the 67325 cells within the terrestrial system.

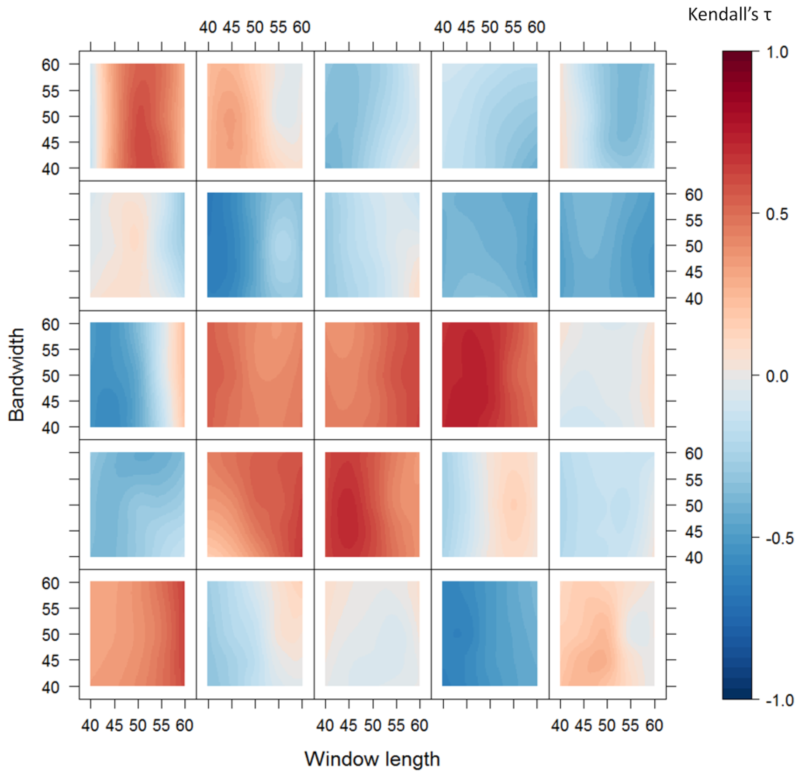

Figure S4. Sensitivity analysis (contourplots) of the temporal trends in the composite indicator represented by the estimated values of Kendall's τ coefficient for a variety of window length and detrending bandwidth for 25 cells randomly chosen among the 32619 cells within the marine system.

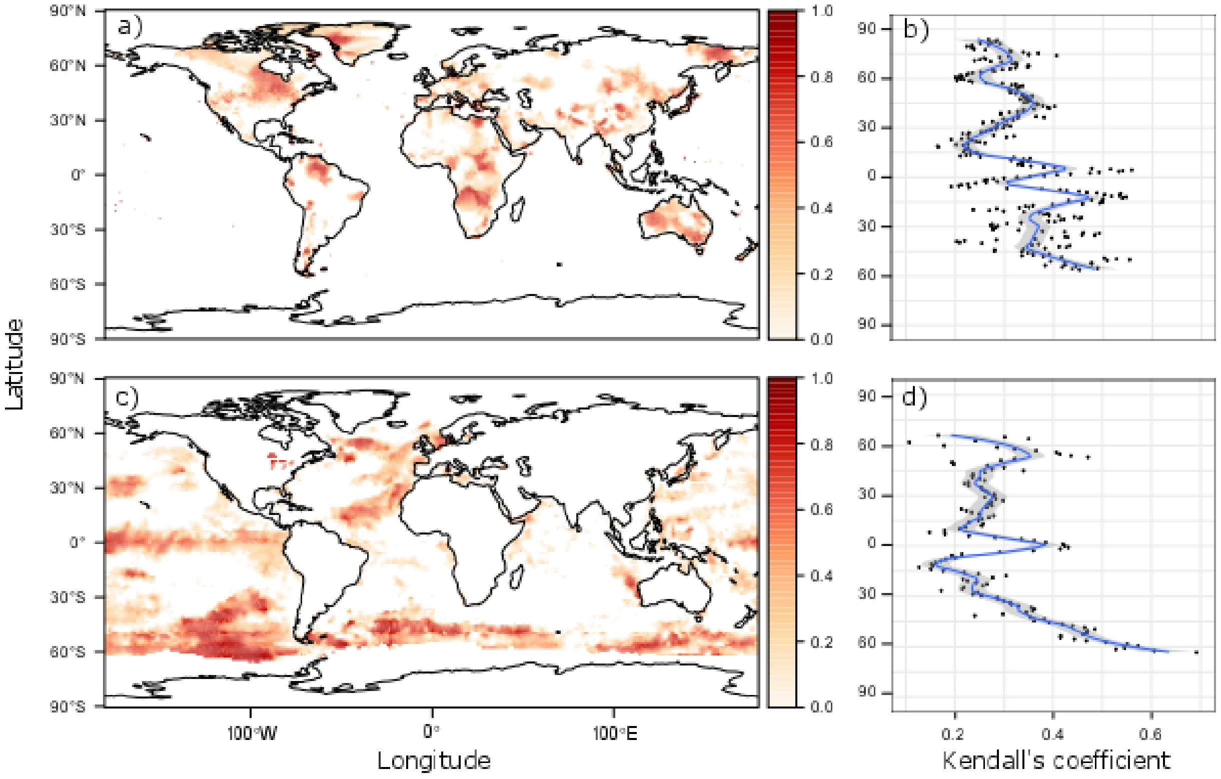

Figure S5. Worldwide trends (Kendall's τ coefficient) and latitudinal gradient in the composite EWI computed without skewness and kurtosis in terrestrial (a) and marine (b) systems. For further details see the legend of Fig. 3 in the main text.

Table S1. Correlations (Pearson's ρ coefficient) between the temporal trends (Kendall's τ rank correlation coefficient) obtained in each pixel for eight leading indicators classically used in the literature and available within the package "earlywarnings". AR1=autoregressive coefficient of AR(1) model; SD=Standard deviation; SK=Skewness; KU=Kurtosis; CV=Coefficient of variation; RR=Return Rate (inverse of AR(1) coefficient); AF=autocorrelation at first lag; DR=Density ratio.

System	EWI	AR1	SD	SK	KU	CV	RR	AF	DR
Terrestrial	AR1	1,000	0,114	0,100	0,005	0,036	0,998	1,000	1,000
	SD	0,114	1,000	0,005	-0,070	-0,017	0,114	0,115	0,115
	SK	0,100	0,005	1,000	0,367	-0,116	0,098	0,100	0,100
	KU	0,005	-0,070	0,367	1,000	-0,027	0,008	0,005	0,005
	CV	0,036	-0,017	-0,116	-0,027	1,000	0,033	0,035	0,035
	RR	0,998	0,114	0,098	0,008	0,033	1,000	0,998	0,998
	AF	1,000	0,115	0,100	0,005	0,035	0,998	1,000	1,000
	DR	1,000	0,115	0,100	0,005	0,035	0,998	1,000	1,000
Marine	AR1	1,000	0,388	-0,024	-0,204	-0,019	1,000	0,999	0,999
	SD	0,388	1,000	-0,191	-0,435	-0,018	0,388	0,411	0,411
	SK	-0,024	-0,191	1,000	0,465	-0,002	-0,024	-0,029	-0,029
	KU	-0,204	-0,435	0,465	1,000	-0,019	-0,204	-0,213	-0,213
	CV	-0,019	-0,018	-0,002	-0,019	1,000	-0,019	-0,023	-0,023
	RR	1,000	0,388	-0,024	-0,204	-0,019	1,000	0,999	0,999
	AF	0,999	0,411	-0,029	-0,213	-0,023	0,999	1,000	1,000
	DR	0,999	0,411	-0,029	-0,213	-0,023	0,999	1,000	1,000

Table S2. Summary of the median of trends *per* terrestrial ecoregions for five EWI classically used in the literature and a composite EWI calculated from individual indicators (for more details see the main text). For indicator abbreviations see Table S1. To provide more details relative to the main text, the biomes were compiled from the World Wildlife Fund (WWF) Terrestrial Ecoregions Of the World (TEOW; http://www.worldwildlife.org/) which provide a classification of biomes at a finer scale relative to the Köppen-Geiger climatic classification.

Terrestrial Ecoregions	AR1	SD	SK	KU	CV	Composite EWI
Boreal Forests/Taiga	-0.133	-0.310	-0.154	-0.162	0.205	-0.184
Deserts and xeric shrublands	0.037	-0.235	-0.027	0.028	0.265	-0.060
Flooded grasslands and savannas	-0.047	-0.307	0.241	-0.054	0.178	-0.124
Mangroves	-0.362	-0.277	0.043	0.011	0.195	-0.231
Mediterranean forests, woodlands and Scrub	0.357	0.260	0.125	0.122	0.134	0.269
Montane grasslands and shrublands	-0.109	-0.254	-0.023	0.054	0.261	-0.039
Temperate broadleaf and mixed forests	0.164	0.011	-0.279	-0.062	0.199	-0.071
Temperate conifer forests	-0.292	-0.521	-0.184	-0.316	0.283	-0.223
Temperate grasslands, savannas and shrublands	0.057	-0.060	-0.471	-0.190	0.251	-0.199
Tropical and subtropical coniferous forests	-0.096	-0.296	0.121	0.153	0.235	-0.012
Tropical and subtropical dry broadleaf forests	-0.356	-0.315	0.155	0.040	0.220	-0.191
Tropical and subtropical grasslands, savannas and shrublands	-0.276	0.147	0.054	-0.012	0.221	0.008
Tropical and subtropical moist broadleaf forests	-0.383	-0.215	0.112	0.030	0.199	-0.144
Tundra	0.450	-0.521	0.218	-0.062	0.216	0.115

Table S3. Summary of the median of trends *per* marine ecoregions for five EWI classically used in the literature and a composite EWI calculated from individual indicators (for more details see the text). For indicator abbreviations see Table S1. As in the main text, the biomes were compiled from the World Wildlife Fund (WWF) Marine Ecoregions Of the World (MEOW).

Marine Ecoregions	AR1	SD	SK	KU	CV	Composite EWI
Arctic	-0.363	-0.554	0.066	0.065	0.090	-0.206
Central Indo-Pacific	0.189	-0.517	0.314	0.238	0.202	0.152
Eastern Indo-Pacific	0.433	-0.239	0.168	-0.107	0.177	0.203
Southern Ocean	0.358	0.352	-0.101	-0.265	0.106	0.040
Temperate Australasia	0.142	-0.085	0.290	0.339	0.011	0.217
Temperate Northern Atlantic	0.090	-0.029	0.069	-0.191	0.206	-0.053
Temperate Northern Pacific	0.035	-0.479	0.139	0.112	0.237	-0.022
Temperate South America	0.242	-0.548	0.386	0.464	0.077	0.367
Temperate Southern Africa	0.020	-0.352	-0.269	-0.460	0.085	-0.390
Tropical Atlantic	-0.221	-0.408	-0.009	-0.133	0.177	-0.203
Tropical Eastern Pacific	0.239	-0.266	0.199	0.458	0.239	0.215
Western Indo-Pacific	0.076	-0.413	0.326	0.500	0.141	0.217

Figure S6. High resolution map for the composite EWS in the terrestrial system

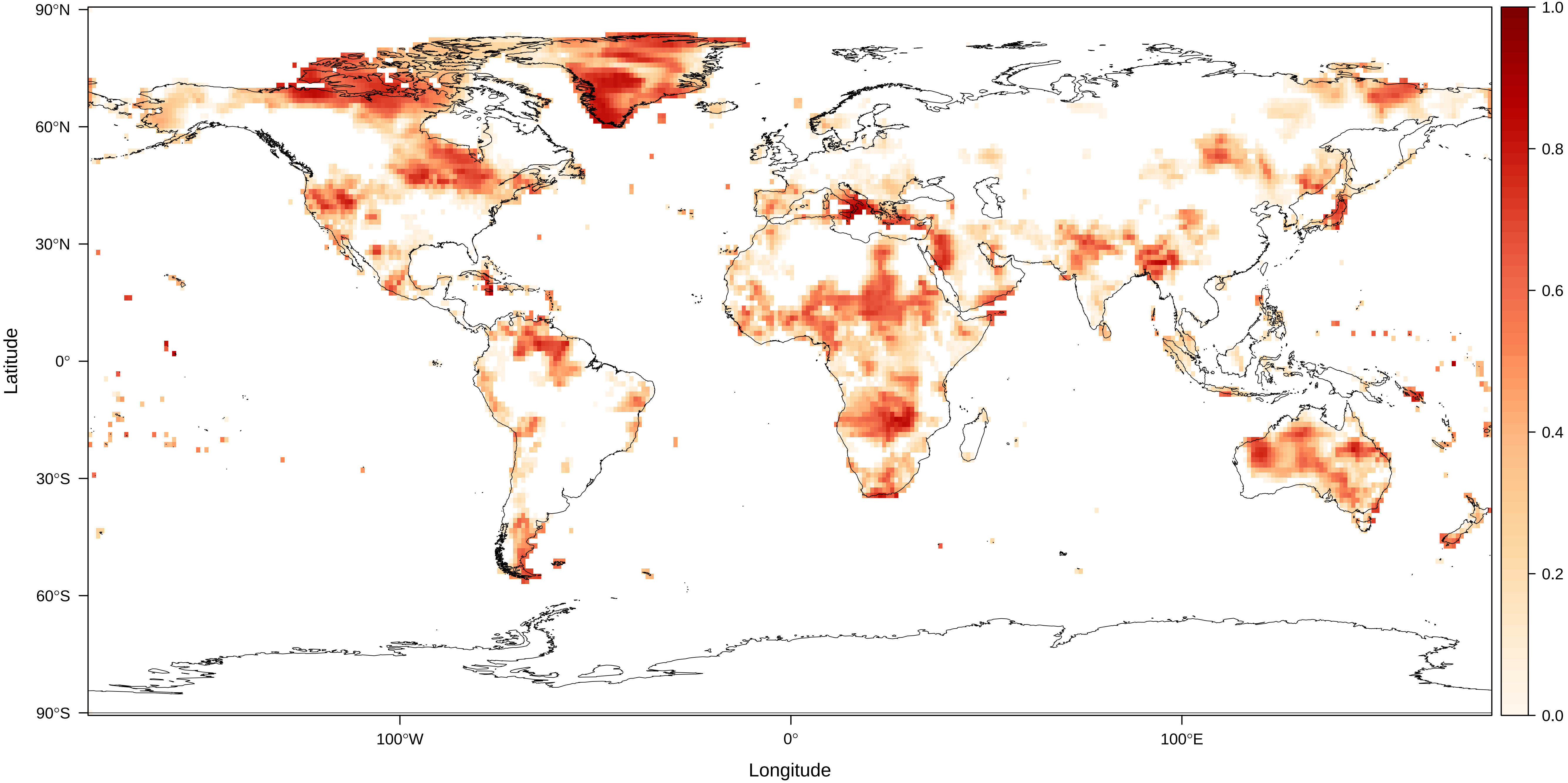

Figure S7. High resolution map for the composite EWS in the marine system

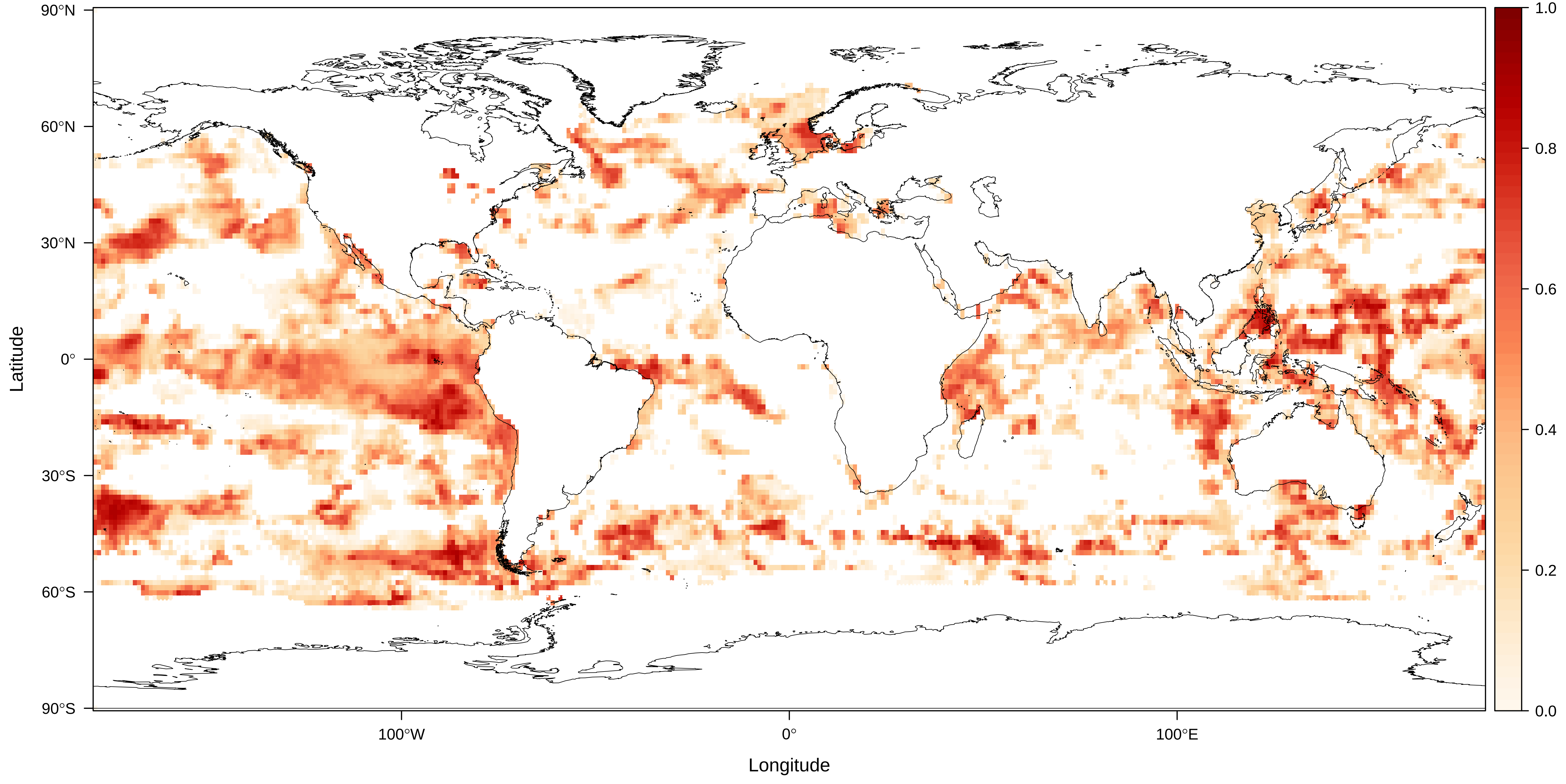

Supplement: Supplementary file 1 — Supplementary information [file 41598_2018_28386_MOESM1_ESM.pdf]
